# Hydrogen/Deuterium Dynamics in Hydroxyl Salts Co_2_(OH)_3_Br/Co_2_(OD)_3_Br Revealed by Muon Spin Relaxation

**DOI:** 10.3390/ma12132135

**Published:** 2019-07-03

**Authors:** Xing-Liang Xu, Xu-Guang Zheng, Isao Watanabe

**Affiliations:** 1 Department of Physics, Zhejiang University of Science and Technology, Hangzhou 310023, China; 2 Department of Physics, Graduate School of Science and Engineering, Saga University, Saga 840-8502, Japan; 3 Department of Physics, Faculty of Science and Engineering, Saga University, Saga 840-8502, Japan; 4 Advanced Meson Science Laboratory, RIKEN, 2-1 Hirosawa, Wako, Saitama 351-0198, Japan

**Keywords:** dielectric response, ferroelectrics, proton dynamics, magnetism, *μ*SR

## Abstract

The temperature-dependent dynamics of the hydrogen/deuterium atoms in geometrically frustrated magnets Co_2_(OH)_3_Br and its deuterated form Co_2_(OD)_3_Br were investigated by muon spin relaxation (*μ*SR). The deuterium atoms in Co_2_(OD)_3_Br were found to be rapidly fluctuating at high temperatures, which should be arising as a quantum atomic effect due to the small mass of deuterium, then they drastically slowed down toward *T_c_* = 250 K where a broad anomaly appeared in the dielectric response, and finally became quasi-static at around 180 K. Meanwhile, the hydrogen atoms in Co_2_(OH)_3_Br also exhibited a two-step slowing at ~240 K and ~180 K, respectively. The revealed properties in Co_2_(OH)_3_Br/Co_2_(OD)_3_Br are reminiscent of relaxor-type ferroelectrics. The present study suggested the effectiveness of the *μ*SR technique on revealing the hydrogen/deuterium (H/D) dynamics in Co_2_(OH)_3_Br/Co_2_(OD)_3_Br. Furthermore, magnetic coupling was found to be existing at high temperatures in this system. This work provides clear evidence to the mechanism of ferroelectric responses in the hydroxyl salts, i.e., the slowing of protons (deuterium ions) is directly related to the newly revealed ferroelectricity.

## 1. Introduction

Ferroelectricity has been widely studied since the first known ferroelectric material, Rochelle salt, was discovered [1]. The mechanisms to generate ferroelectric phase transitions can be categorized as three well-known types. The first one is a displacement type, for example, the common perovskite ferroelectrics such as BaTiO_3_, in which cations shift relative to anions below *T_c_* [2]. An order-disorder one is the second type, and a typical example is NaNO_2_ where polar molecules are randomly oriented above *T_c_*, and align below *T_c_* [3]. A third type of ferroelectricity has been studied in the triangular mixed valence oxide LuFe_2_O_4_, in which this property arises from the polar arrangement of electrons on Fe^3+^, and the electron arrangement comes from charge frustration on a triangular lattice [4].

Recently ferroelectricity was first discovered quite surprisingly in hydroxyl salts Co_2_(OD)_3_Cl and Co_2_(OD)_3_Br, deuterides of Co_2_(OH)_3_Cl and Co_2_(OH)_3_Br, respectively, which belong to the geometrically frustrated magnetic series *M*_2_(OH)_3_*X* (*M* = Cu, Co, Ni, Mn, Fe, etc., *X* = Cl, Br, I) [5,6,7,8], at temperatures 220–230 K higher than their *T*_N_ through an isotope effect [9]. Since Co_2_(OH)_3_Cl and Co_2_(OH)_3_Br showed magnetic phase transitions at low temperatures and strong magnetic couplings even at high temperatures above 400 K [6,7], they can be viewed as a unique series of multiferroic materials, if the ferroelectric transition gets confirmed. Since neither magnetic order nor obvious structure transition exists in this temperature range, the ferroelectricity in the present materials are not caused by a certain type of magnetic ordering such as the magnetic ordering inducing ferroelectricity reported in TbMnO_3_ [10] and *R*CrO_3_ [11], due to the magnetic transition occurring at *T*_N_ = 10.5 K in Co_2_(OD)_3_Cl (*T*_N1_ = 6.2 K and *T*_N2_ = 4.8 K in Co_2_(OD)_3_Br) [9], which is much lower than the ferroelectric transition temperatures. By combining data from muon spin relaxation (*μ*SR) and Raman spectroscopy experiments, we provided clear evidence for the ferroelectric transition mechanism in Co_2_(OD)_3_Cl, wherein a critical slowing of quantum D atoms occurred, suggesting deuterium ordering with a subtle structural transition [12]. In addition, magnetic coupling was also found to be existing below around ~250 K in both Co_2_(OH)_3_Cl and Co_2_(OD)_3_Cl [12]. The present research on Co_2_(OH)_3_Cl/Co_2_(OD)_3_Cl supposes that the ferroelectricity is frustrated [12,13].

In this work, we describe the motion of hydrogen/deuterium (H/D) from the results of *μ*SR measurements performed on Co_2_(OH)_3_Br/Co_2_(OD)_3_Br, providing further evidence to the mechanism of ferroelectric responses in the proton (deuterium)–type system of *M*_2_(OH)_3_*X*, i.e., the slowing of protons (deuterium ions) is directly related to the newly revealed ferroelectricity. In principle, *μ*SR could be applied to proton-type ferroelectrics by studying the dynamics of protons through the fluctuation of their nuclear fields, i.e., the movement of their nuclei.

## 2. Materials and Methods 

The polycrystalline samples of Co_2_(OH)_3_Br and Co_2_(OD)_3_Br were synthesized by the method described in Reference [9]. The dielectric measurements were performed on the two sides of a uniaxially pressed pellet specimen of the polycrystalline powders, using a precision LCR meter over a frequency range of 20 Hz to 100 kHz. *μ*SR experiments were carried out at the RIKEN-RAL Muon Facility of the Rutherford Appleton Laboratory, Didcot, UK, using a double-pulsed positive surface muon beam with the temperature controlled by a standard liquid-He flow-type cryostat in the temperature range between 120 K and 330 K [14]. The powder samples of Co_2_(OH)_3_Br and Co_2_(OD)_3_Br, respectively, were wrapped using the thin aluminum foil and pressed into a pellet with 3 × 3 cm^2^ area and 1 mm thickness, and then tightly covered with a 25-*μ*m thick high purity silver foil to achieve good thermal contact. In the *μ*SR measurement, spin-polarized positive muons (*μ*^+^) were implanted into the sample of interest where they stopped very quickly (~100 ps) at an interstitial site in the lattice. The muons make the Larmor precession by feeling the internal magnetic field, after which they decay into an energetic positron and two neutrinos via a weak interaction with a mean lifetime ≈ 2.2 *μ*s. Parity violation of the weak decay dictates that the decay positron is emitted preferentially in the spin direction of muons [15]. Thus, by measuring the spatial distribution of the decay positrons with the forward (F) and backward (B) detectors around the sample, the ensemble average of the time evolution of muon spin polarization can be extracted via the asymmetry function *A*(*t*), given by A(t)=[F(t)−αB(t)]/[F(t)+αB(t)], where α is an experimental calibration factor reflecting the non-uniform counting efficiency of the forward and backward detectors and is estimated from calibration measurements carried out in the paramagnetic state with a small applied transverse-field (TF) of 20 G. Here, TF means an external magnetic field applied to the material perpendicularly to the initial muon spin polarization [16]. When different muons precess around the local field at different Larmor frequencies, the amplitude of the precession signal decays, i.e., spin relaxation. The frequency and relaxation rate provide information about the local magnetic fields inside the material. Consequently, we can examine how the internal magnetic fields of different materials have affected the muons’ spins by analyzing the *μ*SR spectra. The measurements were performed in zero-field (ZF) and longitudinal-field (LF), which mean a zero external field and an external field of 100 G parallel to the initial muon spin polarization, respectively. The ZF-*μ*SR and LF-*μ*SR data were analyzed simultaneously with MUSRFIT [17].

## 3. Results and Discussion 

### 3.1. Dielectric Measurement

**Figure 1:**
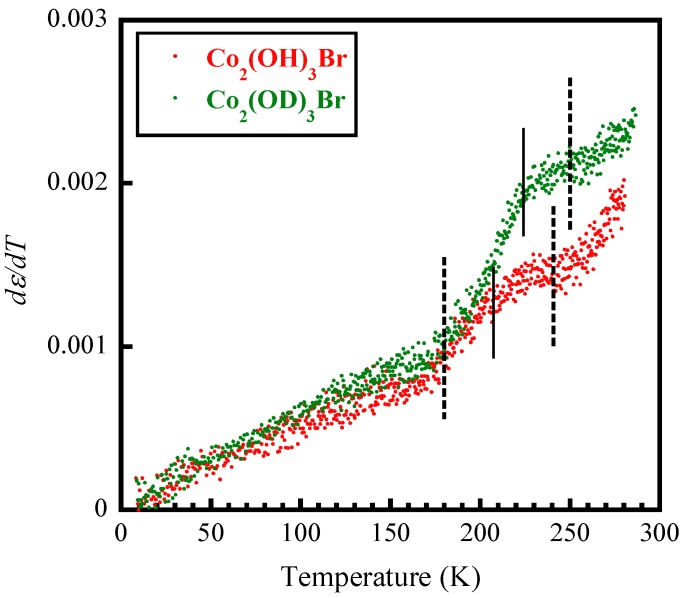
The differential plot of *dε*/*dT−T*, obtained on the pressed-powder pellet samples of Co_2_(OH)_3_Br and Co_2_(OD)_3_Br at 10 kHz. Solid and dashed lines are guides to the eye.

We measured the dielectric constants of pressed-powder pellet samples of Co_2_(OH)_3_Br and Co_2_(OD)_3_Br as a function of temperature and frequency. Because of the loosely-connected particles of the pressed pellet sample, wherein sintering is impossible due to the decomposition of hydroxyl salts, the conventional characterization for ferroelectric materials is not applicable. However, the differential plot of *dε*/*dT*–*T* measured at 10 kHz in the temperature range of 4 K to 295 K as shown in Figure 1, and the frequency-dependent ones in Figure S1 of the Supplemental Material depicted notable changes. After careful inspection, we noted that the changes in Figure 1 should be viewed as broad peaks centered at ~224 K and ~208 K, respectively, for Co_2_(OD)_3_Br and Co_2_(OH)_3_Br (as marked by the thin lines in Figure 1). Meanwhile, these broad peaks extend from ~240–250 K to ~180 K, respectively, for Co_2_(OH)_3_Br and Co_2_(OD)_3_Br. The broad feature and frequency-dependency are reminiscent of relaxor ferroelectrics [18,19], which will be discussed later. These behaviors are much different to those reported for Co_2_(OD)_3_Cl and Co_2_(OH)_3_Cl, wherein the dielectric constant in Co_2_(OD)_3_Cl showed a sharp peak at *T_ε_* = 230 K while in Co_2_(OH)_3_Cl there appeared two individual anomalies near $T^{*}$~210 K and $T^{**}$~160 K, respectively. As reported in Reference [12], ferroelectric transition was verified in Co_2_(OD)_3_Cl. Instead, an incomplete ordering process was suggested for Co_2_(OH)_3_Cl. Considering the similarities and differences between the dielectric changes in Co_2_(OH)_3_Cl/Co_2_(OD)_3_Cl and Co_2_(OH)_3_Br/Co_2_(OD)_3_Br, we suspect that the nature of dielectric responses in Co_2_(OH)_3_Br/Co_2_(OD)_3_Br lies between those of the ferroelectric Co_2_(OD)_3_Cl and the incomplete ordered Co_2_(OH)_3_Cl. This conjecture is reasonable since all these compounds have the same rhombohedral structure in the space group *R*$\bar{3}$m [6,7].

### 3.2. Muon Spin Relaxation (μSR) 

**Figure 2:**
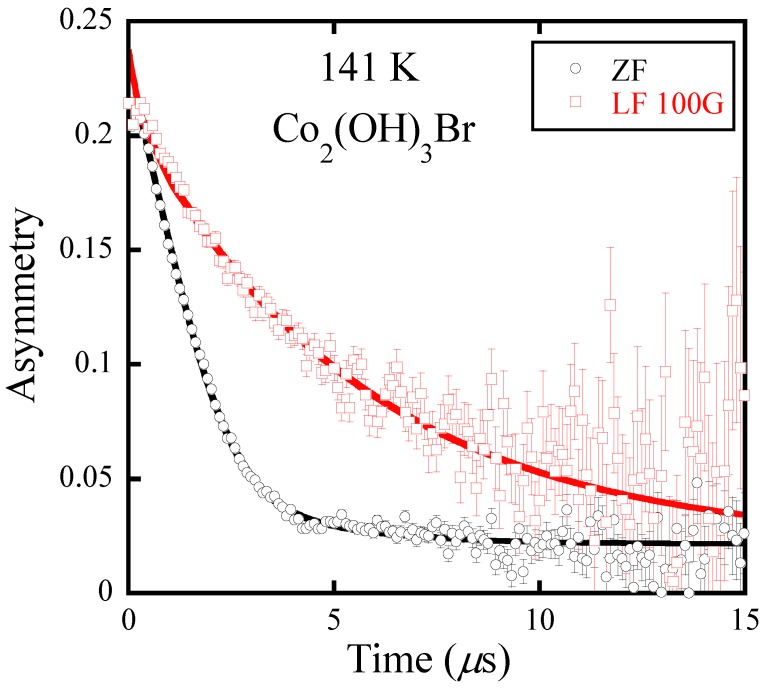
An example of typical ZF- and LF-*μ*SR time spectra for Co_2_(OH)_3_Br measured at 141 K, showing the dynamic of nuclear field and the decoupling of muons with the nuclear relaxation by an external field of 100 G. The solid lines show the best fits according to Equation (1).

Figure 2 shows an example of typical ZF-*μ*SR and LF-*μ*SR spectra for Co_2_(OH)_3_Br obtained at 141 K. Although two successive antiferromagnetic transitions were observed at *T*_N1_ = 6.2 K and *T*_N2_ = 4.8 K, respectively, in Co_2_(OH)_3_Br/Co_2_(OD)_3_Br, the *μ*SR experiments demonstrated magnetic couplings at much higher temperatures [7]. In detail, the ZF-*μ*SR spectrum exhibits the feature of Dynamic Gaussian Kubo-Toyabe (KT) relaxation ($G_{z}^{\mathrm{DKT}}\left(t,\Delta ,\nu \right)$) due to the nuclear fields of H nuclei, and an exponentially relaxing signal caused by magnetic fields due to 3*d* moments of Co^2+^ [20]. The LF-*μ*SR spectrum was measured under a small applied longitudinal magnetic field of 100 G, which seems to be sufficient to quench (decouple) muon spin relaxations by both nuclear dipoles. As a result, the distribution of the nuclear fields of H nuclei and their temperature evolution, i.e., the dynamics of H in the Co_2_(OH)_3_Br lattice at each temperature, can be estimated by analyzing the ZF-*μ*SR and LF-*μ*SR spectra.

**Figure 3:**
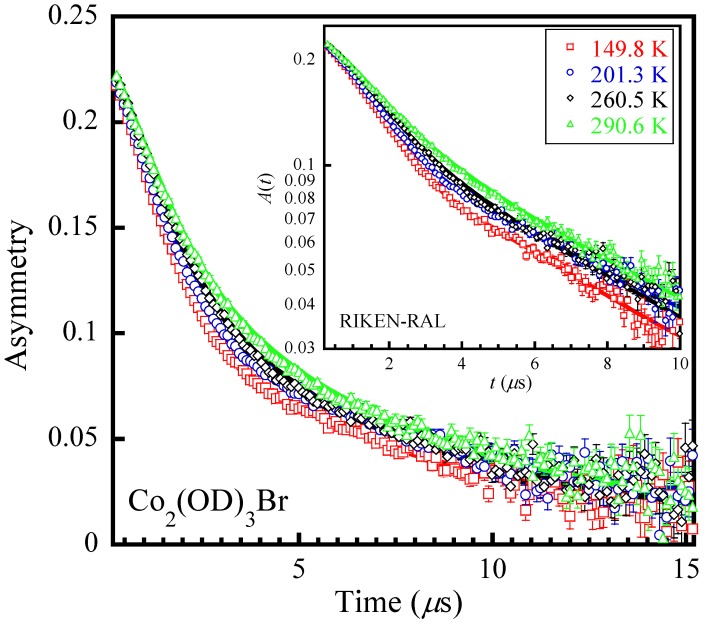
ZF-*μ*SR asymmetries at representative temperatures for Co_2_(OD)_3_Br, showing an evidence of the change in the dynamics of the nuclear fields. The solid thick lines denote the fits using Equation (1). The inset shows the logarithmic plot with the solid lines representing clearly the change in dynamics.

**Figure 4:**
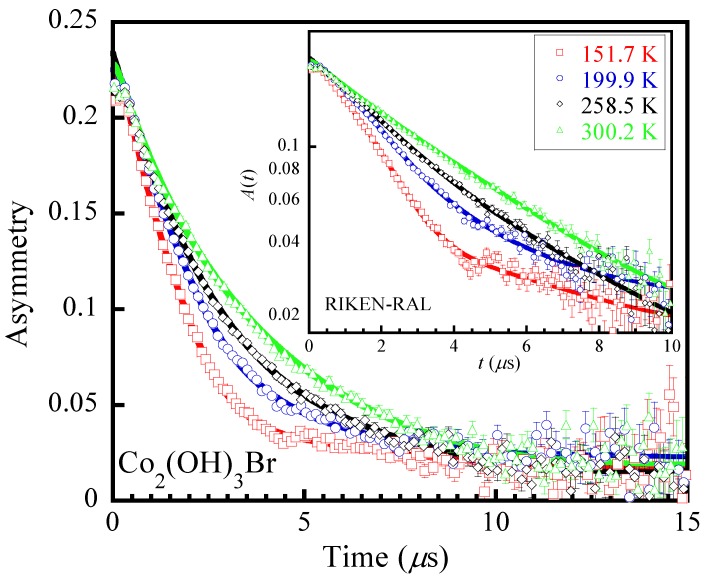
ZF-*μ*SR asymmetries at representative temperatures for Co_2_(OH)_3_Br, showing clear evidence of the change in the dynamics of the nuclear fields. The solid thick lines denote the fits using Equation (1). The inset shows the logarithmic plot with the solid lines representing more clearly the change in dynamics.

Figure 3 and Figure 4 show the ZF-*μ*SR spectra for Co_2_(OD)_3_Br and Co_2_(OH)_3_Br, respectively, at several representative temperatures. These spectra exhibit some understandable changes that suggest changes in the dynamics of the nuclear fields with decreasing temperature. The ZF-*μ*SR and LF-*μ*SR spectra were found to be well fitted simultaneously by a physical model assuming two muon stopping sites near the ${\left[\mathrm{OH}\right]}^{-}/{\left[\mathrm{OD}\right]}^{-}$ and ${\mathrm{Br}}^{-}$, respectively. The full asymmetry is expressed by

(1) $$A\left(t\right)=A_{1}e^{-\lambda_{1}t}G_{z}^{\mathrm{DKT}}\left(t,\Delta ,\nu \right)+A_{2}e^{-\lambda_{2}t}$$

Here, the first term is for the ${\left[\mathrm{OH}\right]}^{-}/{\left[\mathrm{OD}\right]}^{-}$ -site muons given by a combination of an exponentially relaxing signal caused by magnetic fields due to 3*d* moments of Co^2+^, and a Gaussian DKT function $G_{z}^{\mathrm{DKT}}\left(t,\Delta ,\nu \right)$ caused by dynamically fluctuating nuclear moments [20,21], wherein Δ is the line width of the local nuclear field distribution at the disordered sites, and ν represents the field fluctuation rate. The Δ is given by $\Delta =\gamma_{\mu }\sigma$, wherein $\gamma_{\mu }$(=2π × 135.5 MHz/T) is the muon gyromagnetic ratio and σ represents the mean nuclear dipolar field at the muon site. The second term represents the exponential magnetic relaxation for the ${\mathrm{Br}}^{-}$ -site muons. When ν = 0, the DKT function $G_{z}^{\mathrm{DKT}}\left(t,\Delta ,\nu \right)$ becomes a static Gausssian KT function $G_{z}^{\mathrm{KT}}\left(t,\Delta \right)$, which can be simply expressed as $G_{z}^{\mathrm{KT}}\left(t,\Delta \right)=\frac{1}{3}+\frac{2}{3}\left(1-\Delta^{2}t^{2}\right)\exp\left(-\frac{1}{2}\Delta^{2}t^{2}\right)$, as described in Ref. [20]. In Equation (1), *λ*_1_ and *λ*_2_ are the exponential relaxation rates for the two muon stopping sites, respectively. As a matter of fact, an ideal model would have to include the effect of the Co nuclear magnetic fields in the first term in Equation (1), since the ${\left[\mathrm{OH}\right]}^{-}/{\left[\mathrm{OD}\right]}^{-}$ -site muons would also sense the dipolar fields of Co nuclei. However, this kind of function, described in Reference [12], is experimentally unavailable at the present time and technically it is unpractical to perform the fitting using a combination of a KT function multiplied by the DKT function. Consequently Equation (1) is the most appropriate and practical one for the present study. The fitting of a combined function of an exponential with the DKT in Equation (1) can be done by fitting the ZF-*μ*SR and LF-*μ*SR spectra simultaneously using the MUSRFIT program [17].

**Figure 5:**
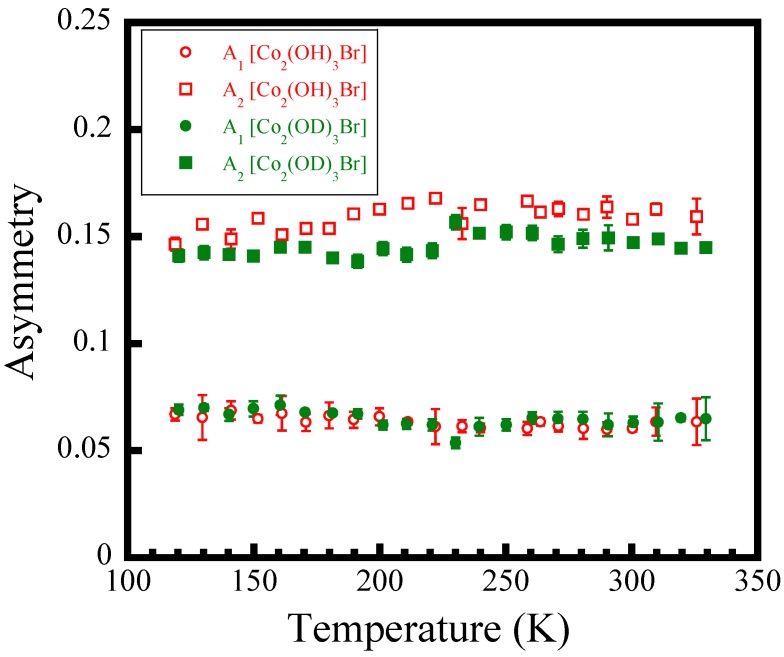
Temperature dependence of asymmetry fractions, $A_{1}$ and $A_{2}$, of the muons’ stopping sites near [OH]^−^/[OD]^−^ and Br^−^, respectively, for Co_2_(OH)_3_Br/Co_2_(OD)_3_Br.

The temperature dependence of *A*_1_ and *A*_2_, which represent the fractions associated with the muons stopping sites near the hydroxyl ${\left[\mathrm{OH}\right]}^{-}/{\left[\mathrm{OD}\right]}^{-}$ and ${\mathrm{Br}}^{-}$ ions, respectively, as shown in Figure 5, are found to be almost constant over the whole measured *T* range, and $A_{1}{/A}_{2}$ ≅ 1/3. This suggests that the distribution of electrostatic potential in the lattice is almost unchanged. As a result, the stability of each muon site is thought to be independent on *T*. It should be noted that muon diffusive behavior often occurs at high temperatures during the *μ*SR measurements performed on hydrogen-bonded ferroelectrics or antiferroelectrics, since the positive muon can be regarded as a light proton with a mass of one ninth of the proton mass [22]. For Co_2_(OH)_3_Br/Co_2_(OD)_3_Br, the thermal activation process due to muon diffusion could be negligible because the ${\left[\mathrm{OH}\right]}^{-}/{\left[\mathrm{OD}\right]}^{-}$- and ${\mathrm{Br}}^{-}$- muon sites fractional occupancies are almost temperature independent. This result provides evidence that Equation (1) which we used is feasible and reasonable for the data analyses.

**Figure 6:**
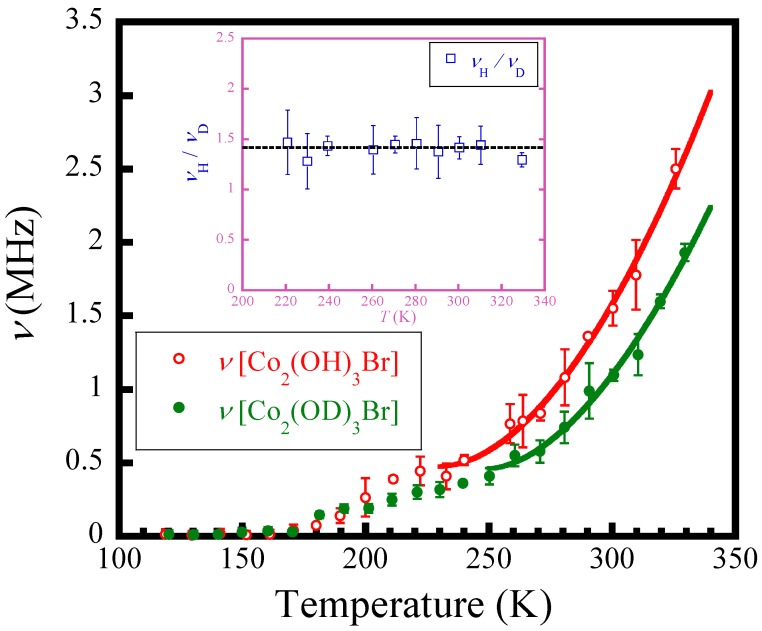
Temperature dependence of the fluctuation rates ν of the nuclear fields. The solid lines represent the fits to the function given by $\nu =\nu_{0}{\left(\frac{T}{T_{c}}-1\right)}^{1.7}$ with *T_c_* = 240 K and 250 K for Co_2_(OH)_3_Br and Co_2_(OD)_3_Br, respectively. Inset: The ratio of ν for the H and D atoms.

The temperature dependence of the field fluctuation rate ν, as given in Figure 6, shows an interesting result. For both Co_2_(OH)_3_Br and Co_2_(OD)_3_Br samples, the general trend of ν showed a prominent two-step decrease with temperature decreasing. In the paraelectric state at high temperatures, the H/D nuclei fluctuate rapidly at a high rate. In detail, for Co_2_(OD)_3_Br, ν rapidly decreased for the first step and showed a slowing with the formula $\nu =\nu_{0}{\left(\frac{T}{T_{c}}-1\right)}^{1.7}$ wherein *T_c_* = 250 K, around which a phase transition most probably occurred at *T_c_*, then decreased slightly with further decreasing *T*, until it reached approximately 0 below around $T^{*}$~180 K. This means that the D atoms were violently fluctuating in the paraelectric phase, which should be arising as a quantum atomic effect due to the small mass of deuterium, then they quickly slowed down toward the phase transition-like temperature ~250 K, and then finally became quasi-static at around 180 K. Meanwhile, the H atoms in Co_2_(OH)_3_Br showed similar behaviors toward *T_c_* = 240 K and $T^{*}$~180 K, respectively. Below ~180 K, both H and D atoms reached a quasi-static state. Here, we should note that one important result shown in the inset of Figure 6, the ratio of ν(*T*) for the H and D atoms in Co_2_(OH)_3_Br and Co_2_(OD)_3_Br, is roughly equal to a square root of their mass ratio expressed by $\frac{\nu_{H}}{\nu_{D}}=\sqrt{\frac{m_{D}}{m_{H}}}=\sqrt{2}$ when *T* ≥ 220 K. This indicates the inexistence of muon diffusive behavior in the paraelectric state, and provides another evidence that Equation (1) is valid for the data analyses. Therefore, the fitted result well describes the dynamics and the slowing behaviors of H/D atoms in Co_2_(OH)_3_Br/Co_2_(OD)_3_Br, and strongly suggests that the H/D atoms are in an unconventional state, i.e., H/D is individually coupled to the tetrahedral other than being bonded to the oxygen behaving as the usual (OH)/(OD) hydroxyl.

As mentioned above, the slowing temperatures of the two-step behavior of H/D atoms obtained from *μ*SR data occurred almost consistent with those of the dielectric constant anomalies for Co_2_(OH)_3_Br/Co_2_(OD)_3_Br, which demonstrates strong correlation between them, i.e., the slowing of protons (deuterium ions) should be responsible for the dielectric response, since there is no pure magnetic ordering or significant lattice change appearing around those temperatures [23,24]. 

The temperature dependence of the exponential relaxation rates *λ*_1_ and *λ*_2_ for Co_2_(OH)_3_Br/Co_2_(OD)_3_Br, respectively, are given in Figure S2 of the Supplemental material. Considering the similar behavior in Co_2_(OH)_3_Cl/Co_2_(OD)_3_Cl, the unusual result of *λ*_1_ can be viewed as the influence from the Co nuclei [12]. The result of *λ*_2_, which purely reflects the magnetic coupling between the Co^2+^ ions, showing a similar manner of monotonically increasing for these compounds in the whole temperature range investigated. This result is consistent with the magnetic susceptibility measurement [7]. In addition, Figure S3 presents the temperature dependence of line widths Δ for Co_2_(OH)_3_Br/Co_2_(OD)_3_Br (see also the Supplemental material). The line widths Δ are almost temperature independent of *T*, suggesting that the model proposed for Co_2_(OH)_3_Br/Co_2_(OD)_3_Br given by Equation (1) has fairly well described the muon spin relaxation in this system. Considering the influence from Co nuclei at the ${\left[\mathrm{OH}\right]}^{-}/{\left[\mathrm{OD}\right]}^{-}$ -site, the Δ values are a bit larger than those expected from the H/D nuclear moments. It also should be noted that the values are almost consistent with those obtained from Co_2_(OH)_3_Cl/Co_2_(OD)_3_Cl [12].

In addition, we attempted to fit the ZF-*μ*SR and LF-*μ*SR spectra for Co_2_(OH)_3_Br and Co_2_(OD)_3_Br using

(2) $$A\left(t\right)=A_{1}e^{-\lambda_{1}t}G_{z}^{\mathrm{DKT}}\left(t,\Delta_{1},\nu \right)+A_{2}e^{-\lambda_{2}t}G_{z}^{\mathrm{KT}}\left(t,\Delta_{2}\right)$$

which was already successfully performed for the data analyses for Co_2_(OH)_3_Cl/Co_2_(OD)_3_Cl [12]. However, λ_2_ was found to be nearly 0 with Equation (2) and such a fit was less reliable than the fit by Equation (1). Therefore, Equation (1) was utilized to fit all the ZF-*μ*SR and LF-*μ*SR spectra simultaneously, and our analysis of the experimental data well described the dynamics behavior of the H and D atoms in Co_2_(OH)_3_Br/Co_2_(OD)_3_Br.

The present *μ*SR results on Co_2_(OH)_3_Br/Co_2_(OD)_3_Br differ significantly from those of Co_2_(OH)_3_Cl/Co_2_(OD)_3_Cl in that a two-step slowing process for the H/D atoms have been observed in the present compounds. As reported in Ref. 12, Co_2_(OD)_3_Cl showed a single critical slowing and a sharp dielectric constant peak at *T_ε_* = 230 K, wherein a ferroelectric phase transition was further verified by Raman spectroscopic studies. Meanwhile, Co_2_(OH)_3_Cl showed one small anomaly at $T^{*}$~210 K and another broad one at $T^{**}$~160 K. In the latter, although the *μ*SR study showed similar critical slowing for the H atoms toward $T^{*}$~210 K, Raman spectroscopic studies strongly suggested a short-range ordering at $T^{*}$~210 K in Co_2_(OH)_3_Cl, followed by the appearance and softening of two modes correlated to the O-H bond below $T^{**}$~160 K. Comparing the similarities and differences in the dielectric and *μ*SR results of Co_2_(OH)_3_Br/Co_2_(OD)_3_Br to those of Co_2_(OH)_3_Cl/Co_2_(OD)_3_Cl, one may reasonably conjecture that the nature of dielectric responses in Co_2_(OH)_3_Br/Co_2_(OD)_3_Br lies between those of the ferroelectric Co_2_(OD)_3_Cl and the incompletely ordered Co_2_(OH)_3_Cl. Both Co_2_(OH)_3_Br/Co_2_(OD)_3_Br and Co_2_(OH)_3_Cl/Co_2_(OD)_3_Cl crystallize in the same rhombohedral structure in the space group *R*$\bar{3}$m [6,7], with slightly longer Co-Br-Co bond lengths in Co_2_(OH)_3_Br/Co_2_(OD)_3_Br than in Co_2_(OH)_3_Cl/Co_2_(OD)_3_Cl. A preliminary Raman spectroscopic study has been performed on Co_2_(OD)_3_Br [25]. Compared with eight new modes observed in Co_2_(OD)_3_Cl, six new vibration modes below ~250 K appeared in Co_2_(OD)_3_Br, implying symmetry breaking similar to that in Co_2_(OD)_3_Cl.

It must be noted that the *μ*SR detects the dynamics of H/D atoms on an atomic scale. The two-step slowing behaviors of the H/D atoms in Co_2_(OH)_3_Br/Co_2_(OD)_3_Br and the continuously-changed broad peak feature in their dielectric constants can be consistently interpreted by the following scenario. For example, in Co_2_(OD)_3_Br, the deuterium atoms drastically slowed down at ~250 K and at the same time became short-range ordered. Then they further slowed down till quasi-static with their correlation length gradually increasing upon cooling. The linear decrease between *T*_c_ and $T^{*}$ in Figure 6 agrees well with this scenario. 

The above scenario reminds us of relaxor-type ferroelectrics. Relaxor ferroelectrics show permanent dipole moment in domains within the nano-length scale [18,19]. The slowing behaviors of the H/D atoms and the broad dielectric peaks in Co_2_(OH)_3_Br/Co_2_(OD)_3_Br can be interestingly explained by relaxor-type ferroelectrics resulting from the ordering of the H/D atoms. As demonstrated in conventional relaxor ferroelectrics, although they have been studied for over fifty years, the mechanism is still not completely understood and is the subject of continuing research. We hope for further studies using single crystals for a more detailed understanding of the ferroelectric-like behavior in this system.

In addition, combined with the fact that magnetic coupling with high temperatures were found in Co_2_(OH)_3_Br/Co_2_(OD)_3_Br, we may therefore suspect that they can be also viewed as a new type of multiferroic, like Co_2_(OD)_3_Cl, since this is a very unexpected phenomenon and the mechanism should be different from that in the classified type-I and type-II multiferroics [26]. Further microscopic studies, for example, of high-quality growth of single crystal samples, is strongly needed to confirm the feature in this system. 

## 4. Conclusions

In conclusion, we have investigated the dielectric response of geometrically frustrated magnet Co_2_(OH)_3_Br and its deuterated Co_2_(OD)_3_Br. Broad peaks centered at ~224 K and ~208 K for Co_2_(OD)_3_Br and Co_2_(OH)_3_Br, respectively, were observed in the dielectric constant measurements. The analyses of *μ*SR measurements show that the deuterium atoms in Co_2_(OD)_3_Br rapidly fluctuate in the paraelectric phase, which should be arising as a quantum atomic effect due to the small mass of deuterium, then drastically slow down toward the dielectric anomaly appearing at *T_c_* = 250 K, and finally became quasi-static at around 180 K. Meanwhile, the hydrogen atoms in Co_2_(OH)_3_Br also exhibited a two-step slowing at ~240 K and ~180 K, respectively. A scenario to link these behaviors in Co_2_(OH)_3_Br/Co_2_(OD)_3_Br to relaxor-type ferroelectrics has been presented. The present study suggested the effectiveness of the *μ*SR technique on revealing the H/D dynamics in Co_2_(OH)_3_Br/Co_2_(OD)_3_Br. In addition, both Co_2_(OH)_3_Br and Co_2_(OD)_3_Br compounds exhibited magnetic coupling with exceptionally high temperatures. This work provides clear evidence to the mechanism of ferroelectric responses in the hydroxyl salts, i.e., the slowing of protons (deuterium ions) is directly related to the newly revealed ferroelectricity.

## Supplementary Materials

The following are available online at https://www.mdpi.com/1996-1944/12/13/2135/s1, Figure S1: Temperature dependence of the *dε*/*dT* for (a) Co_2_(OD)_3_Br and (b) Co_2_(OH)_3_Br, respectively, measured at frequencies of 100, 10, and 1 kHz; Figure S2: Temperature dependence of the exponential relaxation rates *λ*_1_ and *λ*_2_ for Co_2_(OH)_3_Br/Co_2_(OD)_3_Br; Figure S3: The temperature dependence of the nuclear dipolar line widths Δ for Co_2_(OH)_3_Br/Co_2_(OD)_3_Br.
