# Supplementary material for: Hydrogen/Deuterium Dynamics in Hydroxyl Salts Co2(OH)3Br/Co2(OD)3Br Revealed by Muon Spin Relaxation
Source: Materials (Basel). 2019 Jul 3;12(13):2135. doi: 10.3390/ma12132135 (PMC6651803; doi:10.3390/ma12132135)
Supplement: Supplementary file 1 [file materials-12-02135-s001.pdf]

Supplementary Materials: Hydrogen/Deuterium Dynamics in Hydroxyl Salts $Co_2(OH)_3Br/Co_2(OD)_3Br$ Revealed by Muon Spin Relaxation

Xing-Liang Xu, Xu-Guang Zheng and Isao Watanabe

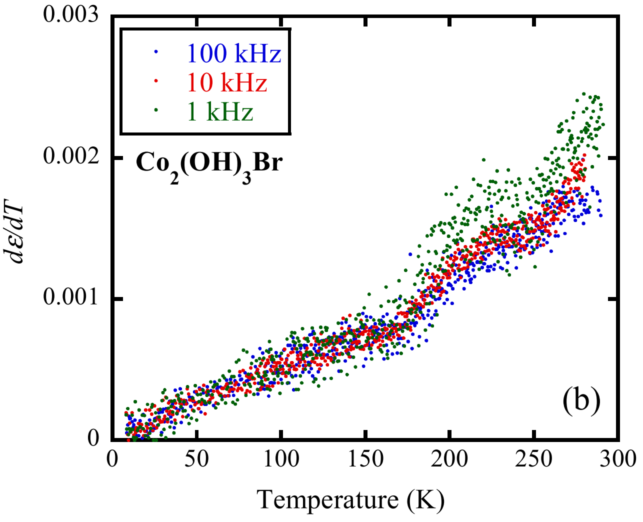

(**a**)

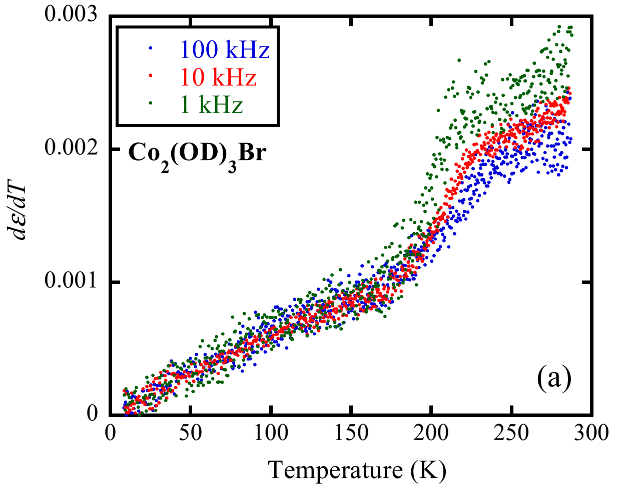

(**b**)

Figure S1: Temperature dependence of the *dε/dT* for (**a**) $Co_2(OD)_3Br$ and (**b**) $Co_2(OH)_3Br$, respectively, measured at frequencies of 100, 10, and 1 kHz.

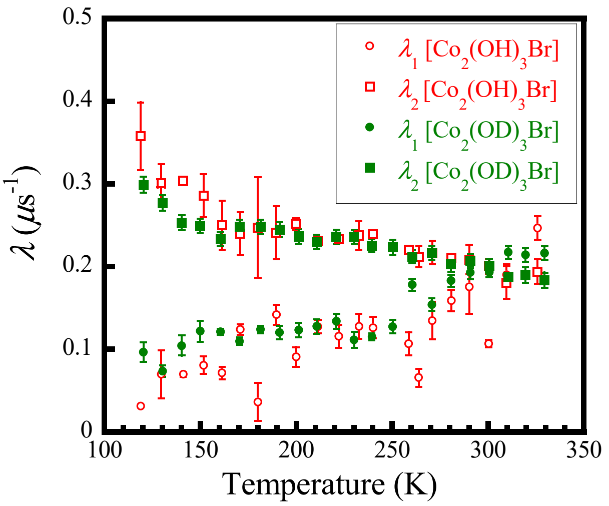

Figure S2: Temperature dependence of the exponential relaxation rates λ_1 and λ_2 for $Co_2(OH)_3Br/Co_2(OD)_3Br$.

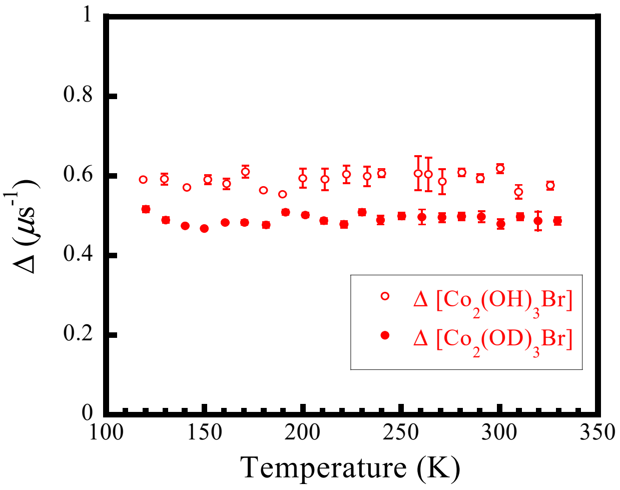

Figure S3: The temperature dependence of the nuclear dipolar line widths Δ for $Co_2(OH)_3Br/Co_2(OD)_3Br$.
